# Species-Specific Responses of Corals to Bleaching Events on Anthropogenically Turbid Reefs on Okinawa Island, Japan, over a 15-year Period (1995–2009)

**DOI:** 10.1371/journal.pone.0060952

**Published:** 2013-04-02

**Authors:** Chuki Hongo, Hiroya Yamano

**Affiliations:** Center for Environmental Biology and Ecosystem Studies, National Institute for Environmental Studies (NIES), 16-2 Onogawa, Tsukuba, Ibaraki, Japan; King Abdullah University of Science and Technology, Saudi Arabia

## Abstract

Coral bleaching, triggered by elevated sea-surface temperatures (SSTs) has caused a decline in coral cover and changes in the abundances of corals on reefs worldwide. Coral decline can be exacerbated by the effects of local stressors like turbidity, yet some reefs with a natural history of turbidity can support healthy and resilient coral communities. However, little is known about responses of coral communities to bleaching events on anthropogenically turbid reefs as a result of recent (post World War II) terrestrial runoff. Analysis of region-scale coral cover and species abundance at 17–20 sites on the turbid reefs of Okinawa Island (total of 79 species, 30 genera, and 13 families) from 1995 to 2009 indicates that coral cover decreased drastically, from 24.4% to 7.5% (1.1%/year), subsequent to bleaching events in 1998 and 2001. This dramatic decrease in coral cover corresponded to the demise of *Acropora* species (e.g., *A. digitifera*) by 2009, when *Acropora* had mostly disappeared from turbid reefs on Okinawa Island. In contrast, Merulinidae species (e.g., *Dipsastraea pallida*/*speciosa*/*favus*) and *Porites* species (e.g., *P. lutea*/*australiensis*), which are characterized by tolerance to thermal stress, survived on turbid reefs of Okinawa Island throughout the period. Our results suggest that high turbidity, influenced by recent terrestrial runoff, could have caused a reduction in resilience of *Acropora* species to severe thermal stress events, because the corals could not have adapted to a relatively recent decline in water quality. The coral reef ecosystems of Okinawa Island will be severely impoverished if *Acropora* species fail to recover.

## Introduction

Coral cover and species diversity on coral reefs have shown dramatic recent declines worldwide, as a consequence of factors that include climate change and numerous other anthropogenic stressors [1]–[3]. During the past three decades, this reduction has led to associated declines in the abundance and diversity of reef fishes and other animals and plants in reef ecosystems, as well as declines in ecological richness and capacities for providing food and medicines to human populations in Indo–Pacific and Caribbean regions [4]–[7].

Worldwide data on long-term and large-scale patterns of coral cover [5], [8]–[11] are controversial. Average coral cover in the Caribbean region declined from 50% to 10% during the period 1977–2001 [5], while another study showed that it has changed very little since the mid-1980s or before [8]. Similarly, large-scale monitoring on the center and south Great Barrier Reef (GBR) indicates that coral cover (averaging 29%) was stable from 1995 to 2009 [9], while coral cover for the whole GBR has decreased from 28.0 to 13.8% (0.53%/year) for 1985–2012 [11].

Knowledge of large-scale and long-term changes in species compositions of corals is essential for understanding processes on coral reefs and for implementing conservation and restoration measures in coral reef ecosystems; this knowledge has significant implications for policy makers. It is particularly important to identify which coral species are most vulnerable to disturbances, and which species are the dominant replacement species in the wake of disturbances.

Elevated sea-surface temperatures (SSTs) have had negative impacts on coral cover and the stability of species compositions on reefs worldwide [12]. Coral bleaching, defined as the loss of a coral's symbiotic dinoflagellates and/or their pigments, is a stress-related response that can be triggered by high SSTs [13]. The majority of the world's coral reefs are also threatened by recent anthropogenic terrestrial runoff [14], on account of factors such as increased dissolved and suspended organic matter, light reduction, and sedimentation [15]. However, some coral communities have been able to flourish despite a long natural history of turbidity, suggesting an ability of some corals to tolerate or adapt to turbid conditions [16]. Furthermore, high turbidity may reduce mortality risk due to thermal stress because feeding on particles at high concentrations may enhance coral energy stores [17]. Consequently, knowledge of the combined impacts of elevated SST events and anthropogenic terrestrial runoff on coral communities is of prime interest.

Here, we report on an analysis of the timing and magnitude of changes in coral cover and species composition on anthropogenically turbid reefs of Okinawa Island after thermal stress events in 1998 and 2001. Monitoring surveys of coral cover and species composition have been employed to study the ecology and biology of the reefs on the island since the mid 1990s. The region-scale data set was compiled between 1995 and 2009 (Table S1). The data derived from permanently placed quadrats provide an unbiased view of change in coral communities.

## Methods

### Okinawa Island

**Figure 1:**
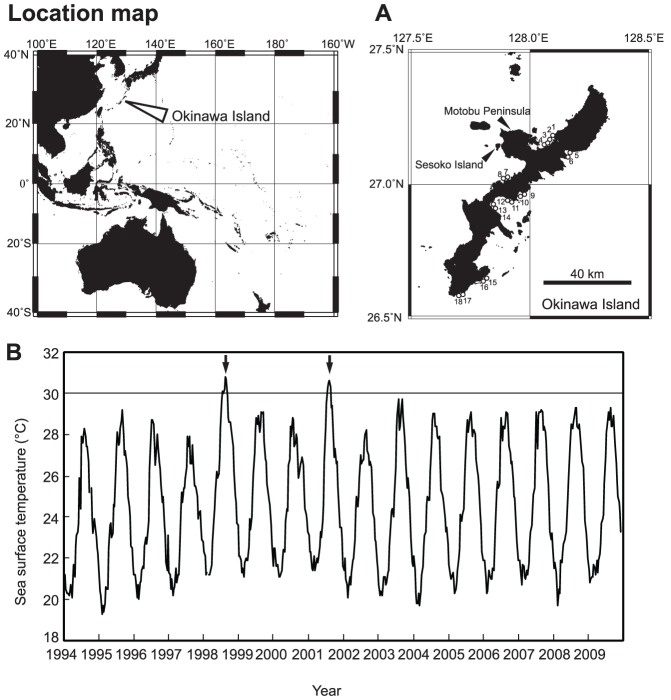
Location of Okinawa Island and monthly means of sea surface temperature (SST) during the period 1994–2009. (A) Okinawa Island, located on the Ryukyu Islands in the Northwest Pacific. Open circles and numbers show the study sites. Location of Sesoko Island characterized by non-turbid reefs is also shown in the figure. (B) Sea surface temperatures were observed at Motobu Peninsula, central Okinawa Island. Data are from the Japan Oceanographic Data Center (http://www.jodc.go.jp/data/coastal/obs_data_index.html) and the Geological Survey of Hokkaido (http://www.gsh.pref.hokkaido.jp/download/temperature_data/index.html). Two thermal stress events, in 1998 and 2001, are indicated by arrows.

Okinawa Island (26°05′N, 127°39′E–26°52′N, 128°15′E) is located in the Ryukyu Islands, Japan, in the western Pacific Ocean (Fig. 1A). The island, which is over 100 km long, is the largest island (1208 km^2^) in the Ryukyu Islands. The island is surrounded by fringing reefs with shallow lagoons a few meters deep. The island is located in the path of the Kuroshio warm current, and is home to more than 300 coral species [18]; the number of species is three times greater than that recorded at similar latitudes on the GBR [19]. The average SST is 21°C in winter and 28°C in summer (Japan Meteorological Agency; see http://www.data.kishou.go.jp/kaiyou/db/kaikyo/ocean/clim/norsst_mon.html). The island was influenced by severe thermal stress (2–3°C higher than in normal years) in the summers of 1998 and 2001 [20] (Fig. 1B). The island has many rivers that discharged into coastal areas and influence the adjacent coral reefs.

Coral reef ecosystems on Okinawa Island have been affected by local and global stresses for the past several decades. Terrestrial runoff related to large-scale agriculture and land-development projects has continuously affected coral reefs on Okinawa Island since World War II [21]. Coral species diversity has been decreasing on the island since the 1970s [22], under the influence of frequent outbreaks of *Acanthaster planci* in the 1970s and 1980s [23] and frequent thermal stress events in the 1980s and 1990s [24]. After 1990, outbreaks of *A. planci* were rarely observed on Okinawa Island [23], but the more recent catastrophic decline of corals was influenced by severe thermal stress events in 1998 and 2001 [19].

### Data on coral cover and species compositions

Our data includes biological and ecological surveys of 17–20 reefs (or sites) conducted in Okinawa prefecture annually from 1995 to 2009 (Table S1). The data represent a compilation of quantitative surveys that measured coral cover and the presence/absence of coral species on reef flats. All surveys used the quadrat method, and the data were collected at permanently placed quadrats (2 m×2 m) on the reefs with total sampling areas of 68–80 m^2^ per site (Table S1).

Corals are one of most difficult taxa to identify at the species level because of morphological polymorphism, intraspecific variation, and phenotypic plasticity [25]. Therefore, careful examination was required during data collection, especially as species were identified by in situ monitoring, without collection of specimens; thus, some mis-identifications are likely to occur in the data. As a result, we combined similar species into species complexes (e.g., *Acropora hyacinthus*/*cytherea* complex, and *Dipsastraea pallida*/*speciosa*/*favus* complex, etc.). To determine which taxa were affected by changes in abundance, we selected 12 dominant genera (*Acropora*, *Stylophora*, *Pocillopora*, *Millepora*, *Stylocoeniella*, *Dipsastraea*, *Favites*, *Goniastrea*, *Leptastrea*, *Montipora*, *Pavona*, and *Porites*) and 12 dominant species or species complexes (*Acropora digitifera*, *A. hyacinthus*/*cytherea*, *A. intermedia*/*muricata*, *Pocillopora damicornis*, *Stylophora pistillata*, *D. pallida*/*speciosa*/*favus*, *Goniastrea aspera*, *Leptastrea purpurea*, *Montipora digitata*, *Oulastrea crispata*, *Porites cylindrica*, and *Porites lutea*/*australiensis*) for further analysis. Detailed results of the analysis are described in the following section.

The non-parametric Kruskal–Wallis test was used to determine whether discernible changes in coral cover (as percent cover) or abundances of genera or species/species complexes occurred through time. The abundance data for each species were compiled in a matrix, with the values “1” and “0” representing “presence” and “absence”, respectively. To calculate the standardized abundance, we averaged the abundance per square meter at each site. The analysis was performed using R software (v. 2.13.0).

### Data on turbidity

The study reefs, which are located near river mouths, are characterized by conditions of continuous turbidity, as represented by suspended particles in sea sediment (SPSS; a parameter used to monitor soil pollution) values in the range of 3–1510 kg/m^3^ [26], equivalent to approximately 0–15 NTUs (Nephelometric Turbidity Units). A rank in SPSS is classified into 9 categories (Rank 1–4, 5a, 5b, 6–8, Table S2) and the rank “5b” represents conditions of turbidity that can start to negatively affect coral cover [21]. A reef in the rank from 6 and 8 is characterized by conditions of high turbidity where only corals tolerant to the stress can survive. The study reefs are generally characterized by the rank between “5b” and “7” (Table S2).

## Results

### Coral cover

**Figure 2:**
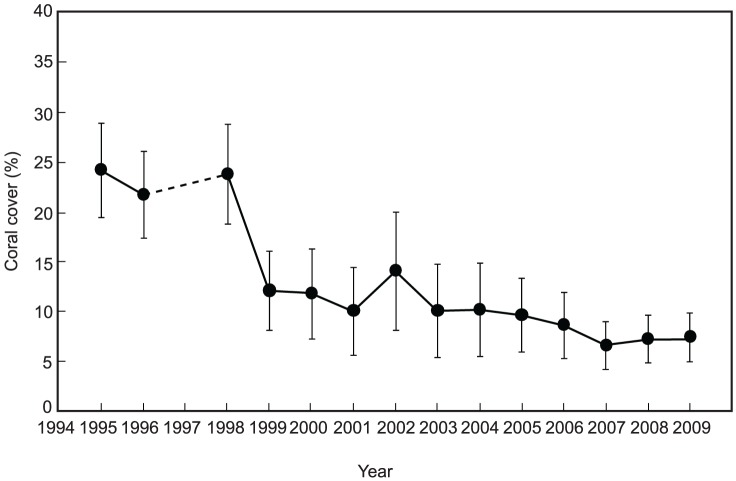
Observed changes in the percent coral cover (mean ± SD) on Okinawa Island reefs from 1995 to 2009 (excluding 1997, during which no surveys were conducted). The number of study sites each year is shown in Table S1.

An analysis of the data from all reefs on Okinawa Island shows that the percentage of coral cover decreased during the period of the study (1995–2009) (Kruskal–Wallis test, *Χ^2^* = 44.1, *P*<0.001). Coral cover declined from 24.4% in 1995 to 12.1% in 1999, and subsequently declined to 7.5% in 2009 (Fig. 2). The rate of decline averaged 1.1%/year from 1995 to 2009.

### Coral genera

**Figure 3:**
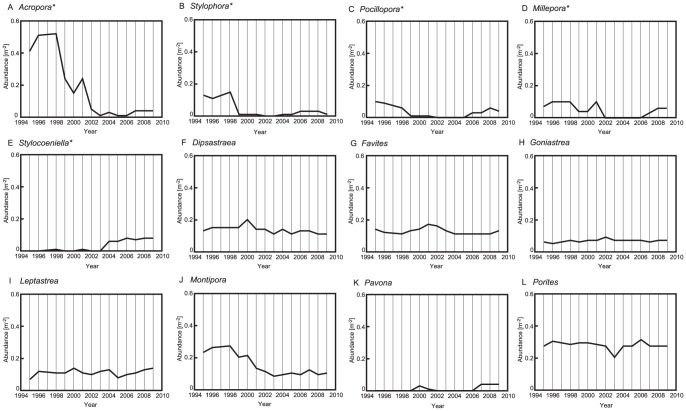
Temporal changes in the abundance of genera (m^−2^) on Okinawa Island during the period 1995–2009. (A): *Acropora*, (B): *Stylophora*, (C): *Pocillopora*, (D): *Millepora*, (E): *Stylocoeniella*, (F): *Dipsastraea*, (G): *Favites*, (H): *Goniastrea*, (I): *Leptastrea*, (J): *Montipora*, (K): *Pavona*, and (L): *Porites*. Significant changes (asterisks) in the abundance were determined by Kruskal–Wallis test. Other genera are shown in Table S3.

We recorded a total of 79 species, 30 genera, and 13 families on the reefs of Okinawa Island (Table S3). Of the genera, some showed significant changes in abundance from 1995 to 2009, whereas others showed no changes in abundance (Fig. 3 and Table S4). The abundance of *Acropora* changed significantly during the study period (Kruskal–Wallis test, *Χ^2^* = 39.1, *P*<0.001; Table S4). Details of temporal trends in the abundance of *Acropora* show two steps of decline: the first severe decline occurred after 1998, and the second occurred after 2001 (Fig. 3A). *Stylophora* also showed significant changes in abundance (Kruskal–Wallis test, *Χ^2^* = 70.6, *P*<0.001), with rapid decreases after 1998 (Fig. 3B). *Pocillopora* and *Millepora* also showed significant changes in abundance during the study period (Kruskal–Wallis test, *Χ^2^* = 36.2, *P*<0.001; *Χ^2^* = 29.8, *P*<0.01, respectively); gradual decreases in the abundances of these genera occurred from 1995 to 2001. Subsequently (by 2005–2006), the abundances of these genera recovered to their previous levels (Fig. 3C, D). *Stylocoeniella* also showed significant changes in abundance (Kruskal–Wallis test, *Χ^2^* = 43.7, *P*<0.001); the genus occurred rarely in the study area from 1995 to 2003, but subsequently re-appeared, starting in 2003 (Fig. 3E). In contrast, there were no significant changes in the abundances of *Dipsastraea*, *Favites*, *Goniastrea*, *Leptastrea*, *Montipora*, *Pavona*, *Porites*, or other genera during the study period (Fig. 3F–L and Table S4).

**Figure 4:**
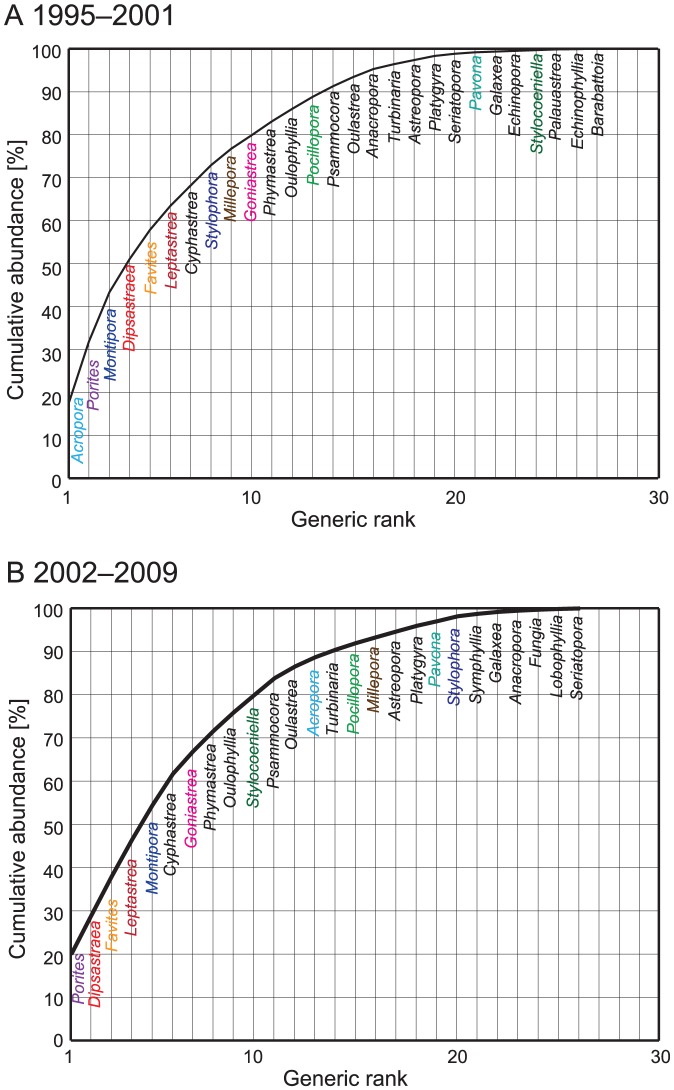
Generic rank in abundance from 1995 to 2001 (A) and the period 2002–2009 (B); a comparison of rankings for the two time periods represents the effects of severe thermal stress events in 1998 and 2001. Abundances for each genus are shown in Table S3. Some notable genera are shown in colored fonts.

Figure 4 shows a change in the ranking of genera between the periods 1995–2001 and 2002–2009, representing before and after states related to two thermal stress events in 1998 and 2001. In terms of relative abundances, *Acropora* declined from the top rank from 1995 to 2001 (abundance of 17.4%) to 13 in rank (abundance of 2.1%) from 2002 to 2009. The rank in abundance of some other genera (*Millepora*, *Montipora*, *Pocillopora*, and *Stylophora*) also declined during this period. In contrast, there was an increase in the rank in abundance for *Porites*, *Pavona*, *Stylocoeniella*, *Leptastrea* and Merulinidae genera (e.g., *Dipsastraea*, *Favites*, and *Goniastrea*) during the period 2002–2009.

### Coral species

**Figure 5:**
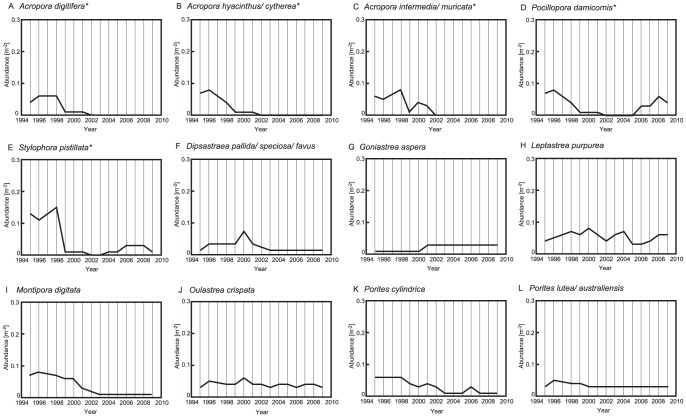
Temporal trends in the abundances of species and species complexes (m−2) on Okinawa Island reefs from 1995 to 2009. (A): *Acropora digitifera*, (B): *Acropora hyacinthus*/*cytherea*, (C): *Acropora intermedia/muricata*, (D): *Pocillopora damicornis*, (E): *Stylophora pistillata*, (F): *Dipsastraea pallida/speciosa/favus*, (G): *Goniastrea aspera*, (H): *Leptastrea purpurea*, (I): *Montipora digitata*, (J): *Oulastrea crispata*, (K): *Porites cylindrica*, and (L): *Porites lutea*/*australiensis*. Significant changes (asterisks) in the abundance were determined by Kruskal–Wallis test. Other species are shown in Table S3.

Some species and species complexes showed significant changes in abundance from 1995 to 2009, whereas others did not (Fig. 5 and Table S5). Some *Acropora* species and species complexes (*A. digitifera*, *A. hyacinthus*/*cytherea*, and *A. intermedia*/*muricata*) show significant changes in abundance through time (Kruskal–Wallis test, *Χ^2^* = 34.2, *P*<0.001; *Χ^2^* = 34.4, *P*<0.001; and *Χ^2^* = 26.8, *P*<0.05, respectively; Table S5); time-series data for these species show decreasing abundances in 1998 and 2001, and the species were nearly absent from the study site in 2009 (Fig. 5A–C). Significant decreases in the abundances of *P. damicornis* and *S. pistillata* (see Kruskal–Wallis test results, Table S5) occurred from 1995 to 2001, but subsequently the species recovered (by 2005–2006) (Fig. 5D and E). Some other *Acropora* species (*Acropora aspera* and *Acropora tenuis*) also showed significant changes in abundances from 1995 to 2009 (Kruskal–Wallis test, *Χ^2^* = 26.2, *P*<0.05; *Χ^2^* = 31.0, *P*<0.01; Table S5), while there were no significant changes in the abundances of other species and species complexes (e.g., *D. pallida*/*speciosa*/*favus*, *G. aspera*, *L. purpurea*, *M. digitata*, *O. crispata*, *P. cylindrica*, and *P. lutea*/*australiensis*) (Fig. 5F–L; Table S5).

**Figure 6:**
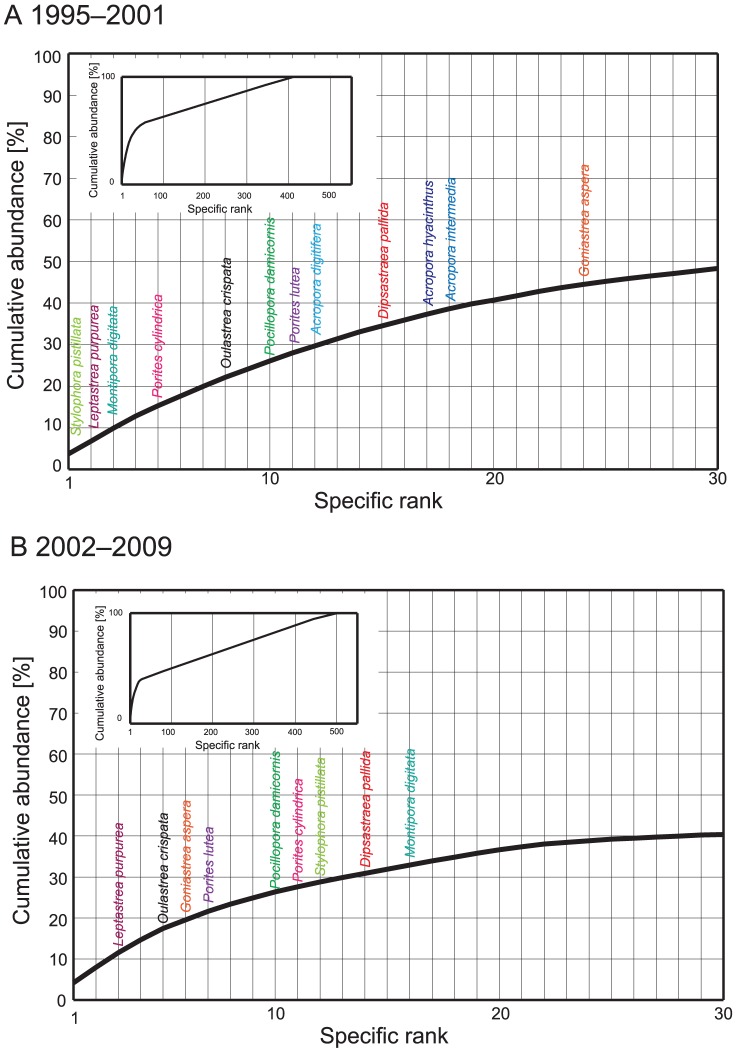
Specific rank in abundance from 1995 to 2001 (A) and from 2002 to 2009 (B); a comparison of rankings for the two time periods represents the effects of severe thermal stress events in 1998 and 2001. Abundances for each species are shown in Table S3. Some notable species are shown in colored fonts. Species rank in abundance for all species (included identified species and spp.) are also shown in the figure.

The ranking of species changed between the periods 1995–2001 and 2002–2009. Some dominant species (e.g., *A. digitifera*, *M. digitata*, and *S. pistillata*) decreased in rank after 2001, while some species (e.g., *G. aspera*, *O. crispata*, and *P. lutea*) increased in rank (Fig. 6).

## Discussion

### Region-scale and long-term decreases in coral cover

Our analyses indicate that coral cover on the anthropogenically turbid reefs of Okinawa Island steadily declined (from 24.4% to 7.5%; 1.1%/year) throughout the study period (1995–2009) and suggest that the reefs may be less resilient to recovery than non-turbid or historically turbid reefs. The bleaching event in 1998 was the largest negative impact on coral cover on the island during the study period, and the bleaching event in 2001 was relatively inconsequential compared to the event in 1998. Although there were no bleaching events after 2001, the coral cover on reefs remained low (less than 10%) after the 2001 event. In contrast, corals from non-turbid reefs on nearby Sesoko Island exhibited greater recovery after the bleaching events [20]. This implies that the anthropogenically turbid reefs of Okinawa have lower resilience, perhaps as a direct consequence of recent terrestrial runoff. Terrestrial runoff generally has a negative impact on larval settlement, juvenile growth, and survival [15].

### Species-specific responses to thermal and turbid stresses

Our dataset indicates that the corals most susceptible to catastrophic declines in abundance in response to recent thermal and turbidity stresses on Okinawa Island are those with tabular and branching morphologies (e.g., *A. aspera*, *A. digitifera*, *A. hyacinthus*/*cytherea*, *A. intermedia*/*muricata*, *A. tenuis*, *P. damicornis*, and *S. pistillata*), but others (e.g., *P. cylindrica* and *M. digitata*) and all massive corals (e.g., *D. pallida*/*speciosa*/*favus*, G. *aspera*, and *P. lutea*/*australiensis*) were relatively unaffected (Table S5). This finding suggests that the effects of the stresses are growth form-specific. Many surveys have demonstrated that, worldwide, tabular and branching corals generally suffer higher rates of mortality than massive corals [20], [27]–[30]. Massive corals are characterized by thick tissue layers that have been hypothesized to have a photoprotective effect on symbiotic dinoflagellates [29]. An expansion and contraction of thick tissues provides a rapid and flexible means of regulating radiant flux reaching zooxanthellae during the thermal stress events and therefore the retracted state of tissue protects the symbionts via self shading [29], [31]. Recent experimental results with sea anemones also support a photoprotective role for thicker host tissues [32]. Moreover, massive corals are generally more resistant to turbid conditions than are tabular and branching corals [17], [33], [34]. In addition to tissue properties, however, the differential susceptibility of corals to thermal and turbidity stresses may be explained by other mechanisms, such as differences in metabolic rate [35], mass-transfer rate [36], stress tolerance among *Symbiodinium* clades [37], [38], and feeding strategy [39].

The branching coral species *P. damicornis* and *S. pistillata* gradually recovered after bleaching events on Okinawa Island probably because they are characterized by unique reproductive strategies. Corals that reproduce by spawning (e.g., species of *Acropora*, *Montipora*, *Dipsastraea*, and *Porites*) release eggs and sperm during a restricted time of year (generally in June on Okinawa Island) [40]. However, *P. damicornis* and *S. pistillata* release planula larvae throughout the year [40], [41]; consequently, abundances of these species could recover quickly in response to disturbance.

**Figure 7:**
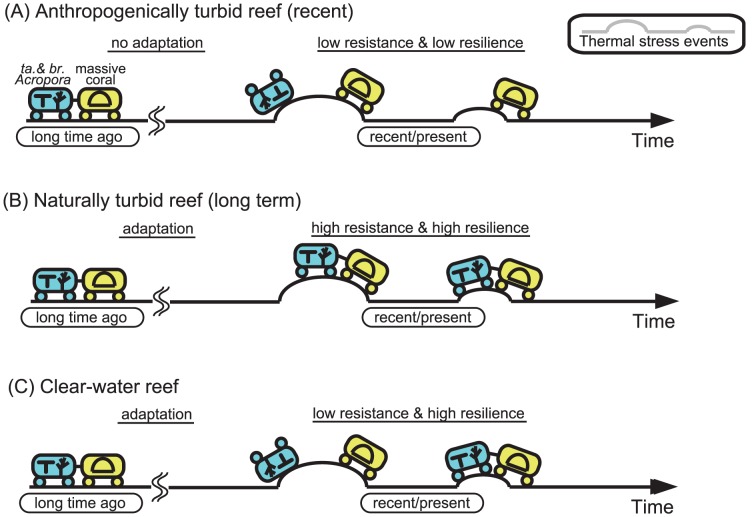
Conceptual model response to thermal stresses on anthropogenically turbid reefs (A), naturally turbid reefs (B), and clear-water reefs (C). Given the result of this study, the model would be most applicable to western Pacific region. (A) Tabular (ta.) and branching (br.) *Acropora* (e.g., *A. digitifera*) from anthropogenically turbid reefs, influenced by recent increasing terrestrial runoff, are intolerant to severe thermal stresses and no recovery as a result of low resistance and low resilience and no adaptation to a condition of turbid. (B) Tabular and branching *Acropora* and massive corals from naturally turbid reefs, adapted to a condition of turbid, are tolerant to severe thermal stresses as a result of high resistance and high resilience. (C) Tabular and branching *Acropora* from clear-water reefs are intolerant to severe thermal stresses as a result of low resistance, but the coral recover the abundance as a result of high resilience. Massive corals are generally tolerant to thermal stresses. Corals are symbolized in coaches in blue (tabular and branching *Acropora*) and in yellow (massive corals). Thermal stresses are symbolized in some slopes in the railway.

High turbidity, influenced by recent (post World War II) terrestrial runoff, could have caused a reduction in resilience of tabular and branching *Acropora* species to severe thermal stress events, because the corals could not have adapted to such a relatively short-term decline in water quality (Fig. 7). Some tabular and branching *Acropora* species on nearby Sesoko Island also declined following the bleaching events, but have shown some signs of recovery since then [20]. This implies that the corals on Sesoko Island have a higher resilience to thermal stress. This is likely related to differences in turbidity between our study reefs and those on Sesoko Island. Our study sites were located near river mouths and have been continuously affected by high turbidity, as a result of recent (post World War II) human activity (e.g., terrestrial runoff related to agriculture and land-development projects), while Sesoko Island is characterized high water transparency. Interestingly, on nearshore reefs of the central GBR, where turbid conditions have been continuous for the past thousands of years [42], some *Acropora* species have continuously survived for a long time and appear to have adapted to turbidity and thermal stresses [16]. Corals on Okinawa Island may have declined in part because they have not yet adapted or acclimatized to the turbid conditions recently developed on the island.

### Decline of *Acropora* species and predicted near-future collapse of reef formations and ecosystems

Given the importance of *Acropora* species to the formation and maintenance of reef ecosystems [43], coral reefs on Okinawa Island and other reefs may become underdeveloped, influenced by the effects of anthropogenic impacts and global warming. Some *Acropora* species (e.g., *A. digitifera*) on non-turbid reefs have recovered after bleaching events [20], [44], but the recent decline in water quality on Okinawa Island and worldwide [16] may delay or prevent the recovery of *Acropora* species. SSTs at Okinawa Island and in other Pacific regions have increased by nearly 1°C in the past 100 years [45], [46], and SSTs will likely increase by at least 2°C during the 21st century [46]. As a result, bleaching is predicted to occur annually or bi-annually on reefs on Okinawa Island and worldwide by 2030–2100 [13], [47], [48]. Furthermore, reefs will also be affected by ocean acidification, resulting in declining calcification rates [2], [49], and by extreme typhoons, resulting in mechanical damage to coral communities [50].

In light of these challenges to reef coral communities, reef conservation planning and restoration efforts are important issues for the maintenance of healthy reef ecosystems. We should reconsider the rankings of vulnerable *Acropora* species on Okinawa Island in the IUCN Red List. *A. digitifera* and *A. hyacinthus* are listed as "Near Threatened" in ver. 2012.2 of the Red List, but *A. intermedia* and *A. muricata*, both species in major decline, are not assessed. Furthermore, we need more knowledge of long-term and large-scale response of corals to local and global stresses in other areas, and further studies into the influence of turbidity on coral resilience on both naturally and anthropogenically turbid reefs.

## Supporting Information

Table S1 Summary of data of coral community from 1995 to 2009 at Okinawa Island.
(DOC)

Table S2 Characteristics of suspended particles in sea sediment (SPSS) on Okinawa Island. Table S2-1: The mean of maximum values of SPSS and the rank from 1995 to 2004 on Okinawa Island. Table S2-2: Relation between the rank in SPSS values and conditions of reef.
(DOC)

Table S3 Temporal change in the coral species from 1995 to 2009 at Okinawa Island.
(DOC)

Table S4 Result of Kruskal–Wallis test of temporal change in the coral genera from 1995 to 2009 at Okinawa Island.
(DOC)

Table S5 Result of Kruskal–Wallis test of temporal change in the coral species from 1995 to 2009 at Okinawa Island. Dominant species of *Acropora*, *Dipsastraea*, *Goniastrea*, *Leptastrea*, *Millepora*, *Montipora*, *Oulastrea*, *Pocillopora*, *Porites*, *Stylophora*, and *Stylocoeniella* were selected for the analysis.
(DOC)
