# Supplementary material for: Species-Specific Responses of Corals to Bleaching Events on Anthropogenically Turbid Reefs on Okinawa Island, Japan, over a 15-year Period (1995–2009)
Source: PLoS One. 2013 Apr 2;8(4):e60952. doi: 10.1371/journal.pone.0060952 (PMC3614915; doi:10.1371/journal.pone.0060952)
Supplement: Table S1 — Summary of data of coral community from 1995 to 2009 at Okinawa Island. (DOC) [file pone.0060952.s001.doc]

| **Survey year** | **Method** | **No. of survey site** | **Sampling area (m^2^)** | **Cited reference** |
| --- | --- | --- | --- | --- |
| 1995 | Quadrat method (2 m × 2 m) | 18 | 72 m^2^ | [1] |
| 1996 | Quadrat method (2 m × 2 m) | 20 | 80 m^2^ | [2] |
| 1998 | Quadrat method (2 m × 2 m) | 18 | 72 m^2^ | [3] |
| 1999 | Quadrat method (2 m × 2 m) | 18 | 72 m^2^ | [4] |
| 2000 | Quadrat method (2 m × 2 m) | 18 | 72 m^2^ | [5] |
| 2001 | Quadrat method (2 m × 2 m) | 18 | 72 m^2^ | [6] |
| 2002 | Quadrat method (2 m × 2 m) | 17 | 68 m^2^ | [7] |
| 2003 | Quadrat method (2 m × 2 m) | 18 | 72 m^2^ | [8] |
| 2004 | Quadrat method (2 m × 2 m) | 18 | 72 m^2^ | [9] |
| 2005 | Quadrat method (2 m × 2 m) | 18 | 72 m^2^ | [10] |
| 2006 | Quadrat method (2 m × 2 m) | 18 | 72 m^2^ | [11] |
| 2007 | Quadrat method (2 m × 2 m) | 18 | 72 m^2^ | [12] |
| 2008 | Quadrat method (2 m × 2 m) | 18 | 72 m^2^ | [13] |
| 2009 | Quadrat method (2 m × 2 m) | 18 | 72 m^2^ | [14] |

[1] Okinawa Prefecture, 1996. Report of fixed-point observation of red soil pollution. Okinawa, Japan.

[2] Okinawa Prefecture, 1997. Report of fixed-point observation of red soil pollution in 1996. Okinawa, Japan.

[3] Okinawa Prefecture, 1999. Report of fixed-point observation of red soil pollution in 1998. Okinawa, Japan.

[4] Okinawa Prefecture, 2000. Report of fixed-point observation of red soil pollution in 1999. Okinawa, Japan.

[5] Okinawa Prefecture, 2001. Report of fixed-point observation of red soil pollution in 2000. Okinawa, Japan.

[6] Okinawa Prefecture, 2002. Report of fixed-point observation of red soil pollution in 2001. Okinawa, Japan.

[7] Okinawa Prefecture, 2003. Report of fixed-point observation of red soil pollution in 2002. Okinawa, Japan.

[8] Okinawa Prefecture, 2004. Report of fixed-point observation of red soil pollution in 2003. Okinawa, Japan.

[9] Okinawa Prefecture, 2005. Report of fixed-point observation of red soil pollution in 2004. Okinawa, Japan.

[10] Okinawa Prefecture, 2006. Report of fixed-point observation of red soil pollution in 2005. Okinawa, Japan.

[11] Okinawa Prefecture, 2007. Report of fixed-point observation of red soil pollution in 2006. Okinawa, Japan.

[12] Okinawa Prefecture, 2008. Report of fixed-point observation of red soil pollution in 2007. Okinawa, Japan.

[13] Okinawa Prefecture, 2009. Report of fixed-point observation of red soil sedimentation in 2008. Okinawa, Japan.

[14] Okinawa Prefecture, 2010. Report of fixed-point observation of red soil sedimentation in 2009. Okinawa, Japan.
