# Supplementary material for: Species-Specific Responses of Corals to Bleaching Events on Anthropogenically Turbid Reefs on Okinawa Island, Japan, over a 15-year Period (1995–2009)
Source: PLoS One. 2013 Apr 2;8(4):e60952. doi: 10.1371/journal.pone.0060952 (PMC3614915; doi:10.1371/journal.pone.0060952)
Supplement: Table S2 — Characteristics of suspended particles in sea sediment (SPSS) on Okinawa Island. Table S2-1: The mean of maximum values of SPSS and the rank from 1995 to 2004 on Okinawa Island. Table S2-2: Relation between the rank in SPSS values and conditions of reef. (DOC) [file pone.0060952.s002.doc]

| Site* | SPSS_max_ (kg/m^3^) | Rank in SPSS** |
| --- | --- | --- |
| 1 | 37.8 | 5b |
| 2 | 303.3 | 7 |
| 3 | 47.9 | 5b |
| 4 | 71.9 | 6 |
| 5 | 244.4 | 7 |
| 6 | 604.7 | 8 |
| 7 | 21.2 | 5a |
| 8 | 19.6 | 5a |
| 9 | 58.8 | 6a |
| 10 | 344.6 | 7 |
| 11 | 56.4 | 6 |
| 12 | 49.0 | 5b |
| 13 | 143.1 | 6 |
| 14 | 132.5 | 6 |
| 15 | 70.1 | 6 |
| 16 | 60.6 | 6 |
| 17 | 27.3 | 5a |
| 18 | 39.0 | 5a |

Table S2-1.

Location of sites (*) are shown in Fig. 1. Ranks in SPSS (**) are shown in Table S2-2.

Data: Kinjo K, Higa E, Uehara M (2005) Transition of sediment of red soil on seabed and its impact on corals. Newsletter of Okinawa Prefectural Institute of Health and Environment 39: 63–74.

| Rank | SPSS (kg/m^3^) | Description |
| --- | --- | --- |
| 1 | 0.4 or less | Below determination limit. Fine particles are not suspended even when bottom sediment is stirred. White sand extends the area and organisms are rarely observed. |
| 2 | 0.4–1 | Fine particles are not easily suspended even when bottom sediment is stirred. White sand extends the area and organisms are rarely observed. |
| 3 | 1–5 | Fine particles can be suspended by stirring the bottom sediment. A lively coral reef ecosystem can be observed. Water transparency is high. |
| 4 | 5–10 | Water becomes little turbid when bottom sediment was stirred, although it will not occur in calm conditions. A lively coral reef ecosystem can be observed. |
| 5a | 10–30 | Fine particles can be observed on the surface of bottom sediment by careful observation. This rank corresponds to the SPSS upper bound where lively coral reef ecosystem can be observed. |
| 5b | 30–50 | Dust-like fine particles cover the sediment surface. The transparency declines and adverse effect on coral coverage starts to occur. |
| 6 | 50–200 | Sediments such as red soil can be seen at a glance. If the bottom sediment is stirred, suspension is apparent in dark color. Above this rank, it is judged that obvious pollution by the man-caused outflow of red soil occurs. |
| 7 | 200–400 | Shoe sole's patterns are left clearly on the surface. Sedimentation of red soil is remarkable, but sand can still be seen. Large colonies of branching *Acropora* will are not seen, and massive corals increases. |
| 8 | 400 or more | Feet get bogged in mud. Sand can rarely be observed. A massive type corals which have strong sediment-resistance distributes. |

Table S2-2.

Data: Omija T (2004) Terrestrial inflow of soils and nutrients. In: The Japanese Coral Reef Society, Ministry of the Environment, editors. Tokyo: Ministry of the Environment. pp. 64–68.
