# Supplementary material for: Species-Specific Responses of Corals to Bleaching Events on Anthropogenically Turbid Reefs on Okinawa Island, Japan, over a 15-year Period (1995–2009)
Source: PLoS One. 2013 Apr 2;8(4):e60952. doi: 10.1371/journal.pone.0060952 (PMC3614915; doi:10.1371/journal.pone.0060952)
Supplement: Table S3 — Temporal change in the coral species from 1995 to 2009 at Okinawa Island. (DOC) [file pone.0060952.s003.doc]

| **Family** | **Genus** | **species** | 1995 | 1996 | 1998 | 1999 | 2000 | 2001 | 2002 | 2003 | 2004 | 2005 | 2006 | 2007 | 2008 | 2009 |
| --- | --- | --- | --- | --- | --- | --- | --- | --- | --- | --- | --- | --- | --- | --- | --- | --- |
| **Acroporidae** |  | **Total** | **0.7** | **0.8** | **0.8** | **0.7** | **0.4** | **0.4** | **0.2** | **0.1** | **0.2** | **0.2** | **0.1** | **0.2** | **0.2** | **0.2** |
|  | ***Acropora*** | **Total** | **0.41** | **0.51** | **0.52** | **0.24** | **0.15** | **0.24** | **0.05** | **0.01** | **0.03** | **0.01** | **0.01** | **0.04** | **0.04** | **0.04** |
|  |  | *Acropora acuminata* | - | - | 0.01 | 0.01 | - | 0.01 | - | - | - | - | - | - | - | - |
|  |  | *Acropora aspera* | - | - | 0.03 | - | - | - | - | - | - | - | - | - | - | - |
|  |  | *Acropora austera* | 0.01 | - | - | - | - | 0.01 | - | - | - | - | - | - | - | - |
|  |  | *Acropora brueggemanni* | - | 0.01 | 0.01 | - | - | - | - | - | - | - | - | - | - | - |
|  |  | *Acropora clathrata* | - | - | 0.01 | - | - | - | - | - | - | - | - | - | - | - |
|  |  | *Acropora cytherea* | 0.03 | 0.03 | 0.01 | - | - | - | - | - | - | - | - | - | - | - |
|  |  | *Acropora digitifera* | 0.04 | 0.06 | 0.06 | 0.01 | 0.01 | 0.01 | - | - | - | - | - | - | - | - |
|  |  | *Acropora divaricata* | - | - | - | - | - | 0.01 | - | - | - | - | - | - | - | - |
|  |  | *Acropora florida* | 0.03 | 0.03 | 0.03 | - | - | - | - | - | - | - | - | - | - | - |
|  |  | *Acropora gemmifera* | 0.01 | 0.01 | - | 0.01 | - | 0.01 | - | - | - | - | - | - | - | - |
|  |  | *Acropora humilis* | 0.03 | 0.03 | 0.01 | - | - | - | - | - | - | - | - | - | - | - |
|  |  | *Acropora hyacinthus* | 0.04 | 0.05 | 0.03 | 0.01 | 0.01 | 0.01 | - | - | - | - | - | - | - | - |
|  |  | *Acropora intermedia* | 0.04 | 0.04 | 0.04 | - | 0.01 | 0.01 | - | - | - | - | - | - | - | - |
|  |  | *Acropora loripes* | - | - | - | - | - | 0.01 | - | - | - | - | - | - | - | - |
|  |  | *Acropora microphthalma* | 0.03 | 0.03 | 0.04 | - | - | 0.01 | - | - | - | - | - | - | - | - |
|  |  | *Acropora monticulosa* | 0.01 | - | - | - | - | 0.01 | - | - | - | - | - | - | - | - |
|  |  | *Acropora muricata* | 0.01 | 0.01 | 0.04 | 0.01 | 0.03 | 0.01 | - | - | - | - | - | - | - | - |
|  |  | *Acropora nana* | - | - | - | 0.04 | - | 0.01 | - | - | - | - | - | - | - | - |
|  |  | *Acropora nasuta* | 0.03 | 0.04 | 0.04 | - | 0.03 | 0.03 | - | - | - | - | - | - | - | - |
|  |  | *Acropora secale* | - | 0.01 | 0.01 | - | 0.01 | - | - | - | - | - | - | - | - | - |
|  |  | *Acropora selago* | - | 0.01 | 0.01 | - | - | - | - | - | - | - | - | - | - | - |
|  |  | *Acropora subulata* | - | - | - | 0.01 | - | 0.01 | - | 0.01 | - | - | - | - | - | - |
|  |  | *Acropora tenuis* | 0.03 | 0.05 | 0.04 | 0.01 | - | - | - | - | - | - | - | - | - | - |
|  |  | *Acropora valida* | 0.01 | 0.01 | - | - | - | - | - | - | - | - | - | - | - | - |
|  |  | *Acropora yongei* | - | - | - | - | - | 0.01 | - | - | - | - | - | - | - | - |
|  |  | other species | 0.04 | 0.09 | 0.07 | 0.10 | 0.04 | 0.01 | 0.05 | - | 0.03 | 0.01 | 0.01 | 0.04 | 0.04 | 0.04 |
|  |  |  |  |  |  |  |  |  |  |  |  |  |  |  |  |  |
|  | ***Anacropora*** | **Total** | **0.01** | **0.01** | **0.01** | **0.19** | **0.00** | **0.00** | **0.00** | **0.00** | **0.01** | **0.01** | **0.00** | **0.00** | **0.00** | **0.01** |
|  |  | *Anacropora* sp. | 0.01 | 0.01 | 0.01 | 0.19 | - | - | - | - | 0.01 | 0.01 | - | - | - | 0.01 |
|  |  |  |  |  |  |  |  |  |  |  |  |  |  |  |  |  |
|  | ***Astreopora*** | **Total** | **0.03** | **0.05** | **0.01** | **0.01** | **0.01** | **0.01** | **0.02** | **0.02** | **0.01** | **0.01** | **0.01** | **0.01** | **0.03** | **0.03** |
|  |  | *Astreopora myriophthalma* | - | - | - | - | - | - | - | - | - | - | - | - | 0.01 | 0.01 |
|  |  | other species | 0.03 | 0.05 | 0.01 | 0.01 | 0.01 | 0.01 | 0.02 | 0.02 | 0.01 | 0.01 | 0.01 | 0.01 | 0.01 | 0.01 |
|  |  |  |  |  |  |  |  |  |  |  |  |  |  |  |  |  |
|  | ***Montipora*** | **Total** | **0.24** | **0.27** | **0.28** | **0.21** | **0.22** | **0.14** | **0.12** | **0.09** | **0.10** | **0.11** | **0.10** | **0.13** | **0.10** | **0.11** |
|  |  | *Montipora aequituberculata* | 0.04 | 0.05 | 0.04 | 0.04 | 0.04 | 0.04 | 0.02 | 0.01 | 0.01 | 0.01 | 0.01 | 0.01 | 0.01 | 0.01 |
|  |  | *Montipora altasepta* | - | - | - | - | - | - | - | - | - | - | - | - | - | - |
|  |  | *Montipora cactus* | - | - | 0.01 | 0.01 | 0.01 | 0.01 | 0.02 | 0.01 | 0.01 | 0.01 | 0.01 | 0.01 | - | - |
|  |  | *Montipora digitata* | 0.07 | 0.08 | 0.07 | 0.06 | 0.06 | 0.03 | 0.02 | 0.01 | 0.01 | 0.01 | 0.01 | 0.01 | 0.01 | 0.01 |
|  |  | *Montipora hispida* | - | - | 0.01 | 0.01 | 0.01 | 0.01 | 0.02 | 0.01 | 0.01 | 0.01 | 0.01 | 0.01 | 0.01 | 0.01 |
|  |  | *Montipora informis* | - | - | - | - | - | - | - | 0.01 | - | - | - | - | - | - |
|  |  | *Montipora samarensis* | - | - | - | - | - | - | - | - | - | - | - | - | - | - |
|  |  | *Montipora stellata* | 0.01 | 0.01 | 0.01 | 0.01 | 0.01 | - | - | - | - | - | - | - | - | - |
|  |  | other species | 0.11 | 0.13 | 0.13 | 0.07 | 0.08 | 0.04 | 0.06 | 0.04 | 0.04 | 0.06 | 0.04 | 0.07 | 0.06 | 0.07 |
|  |  |  |  |  |  |  |  |  |  |  |  |  |  |  |  |  |
| **Agariciidae** |  | **Total** | **0.00** | **0.00** | **0.00** | **0.00** | **0.03** | **0.01** | **0.00** | **0.00** | **0.00** | **0.00** | **0.00** | **0.04** | **0.04** | **0.04** |
|  | ***Pavona*** | **Total** | **0.00** | **0.00** | **0.00** | **0.00** | **0.03** | **0.01** | **0.00** | **0.00** | **0.00** | **0.00** | **0.00** | **0.04** | **0.04** | **0.04** |
|  |  | *Pavona cactus* | - | - | - | - | - | - | - | - | - | - | - | 0.01 | 0.01 | 0.01 |
|  |  | *Pavona decussata* | - | - | - | - | 0.01 | - | - | - | - | - | - | - | - | - |
|  |  | *Pavona divaricata* | - | - | - | - | - | - | - | - | - | - | - | 0.01 | 0.01 | 0.01 |
|  |  | *Pavona frondifera* | - | - | - | - | 0.01 | 0.01 | - | - | - | - | - | - | - | - |
|  |  | *Pavona venosa* | - | - | - | - | - | - | - | - | - | - | - | 0.01 | - | - |
|  |  | other species | - | - | - | - | - | - | - | - | - | - | - | - | 0.01 | 0.01 |
|  |  |  |  |  |  |  |  |  |  |  |  |  |  |  |  |  |
| **Astrocoeniidae** | | **Total** | **0.00** | **0.00** | **0.01** | **0.00** | **0.00** | **0.01** | **0.00** | **0.00** | **0.06** | **0.06** | **0.08** | **0.07** | **0.08** | **0.08** |
|  | ***Stylocoeniella*** | **Total** | **0.00** | **0.00** | **0.01** | **0.00** | **0.00** | **0.01** | **0.00** | **0.00** | **0.06** | **0.06** | **0.08** | **0.07** | **0.08** | **0.08** |
|  |  | *Stylocoeniella guentheri* | - | - | - | - | - | - | - | - | 0.01 | 0.01 | 0.03 | 0.01 | 0.01 | 0.01 |
|  |  | other species | - | - | 0.01 | - | - | 0.01 | - | - | 0.04 | 0.04 | 0.06 | 0.06 | 0.07 | 0.07 |
|  |  |  |  |  |  |  |  |  |  |  |  |  |  |  |  |  |
| **Dendrophylliidae** | | **Total** | **0.00** | **0.01** | **0.01** | **0.04** | **0.03** | **0.04** | **0.03** | **0.01** | **0.00** | **0.03** | **0.03** | **0.03** | **0.03** | **0.03** |
|  | ***Turbinaria*** | **Total** | **0.00** | **0.01** | **0.01** | **0.04** | **0.03** | **0.04** | **0.03** | **0.01** | **0.00** | **0.03** | **0.03** | **0.03** | **0.03** | **0.03** |
|  |  | *Turbinaria irregularis* | - | 0.01 | 0.01 | 0.01 | 0.01 | 0.01 | 0.02 | 0.01 | - | 0.01 | 0.01 | 0.01 | 0.01 | 0.01 |
|  |  | *Turbinaria reniformis* | - | - | - | 0.01 | - | 0.01 | - | - | - | - | - | - | - | - |
|  |  | other species | - | - | - | 0.01 | 0.01 | 0.01 | 0.02 | - | - | 0.01 | 0.01 | 0.01 | 0.01 | 0.01 |
|  |  |  |  |  |  |  |  |  |  |  |  |  |  |  |  |  |
| **Euphylliidae** |  | **Total** | **0.00** | **0.00** | **0.00** | **0.14** | **0.01** | **0.01** | **0.00** | **0.00** | **0.00** | **0.01** | **0.01** | **0.01** | **0.01** | **0.01** |
|  | ***Galaxea*** | **Total** | **0.00** | **0.00** | **0.00** | **0.14** | **0.01** | **0.01** | **0.00** | **0.00** | **0.00** | **0.01** | **0.01** | **0.01** | **0.01** | **0.01** |
|  |  | *Galaxea fascicularis* | - | - | - | 0.14 | 0.01 | 0.01 | - | - | - | 0.01 | 0.01 | 0.01 | 0.01 | 0.01 |
|  |  |  |  |  |  |  |  |  |  |  |  |  |  |  |  |  |
| **Fungiidae** |  | **Total** | **0.00** | **0.00** | **0.00** | **0.00** | **0.00** | **0.00** | **0.00** | **0.00** | **0.00** | **0.00** | **0.01** | **0.00** | **0.00** | **0.01** |
|  | ***Fungia*** | **Total** | **0.00** | **0.00** | **0.00** | **0.00** | **0.00** | **0.00** | **0.00** | **0.00** | **0.00** | **0.00** | **0.01** | **0.00** | **0.00** | **0.01** |
|  |  | *Fungia* sp.1 | - | - | - | - | - | - | - | - | - | - | 0.01 | - | - | - |
|  |  | *Fungia* sp.2 | - | - | - | - | - | - | - | - | - | - | - | - | - | 0.01 |
|  |  |  |  |  |  |  |  |  |  |  |  |  |  |  |  |  |
| **Lobophylliidae** |  | **Total** | **0.00** | **0.00** | **0.00** | **0.00** | **0.01** | **0.00** | **0.00** | **0.00** | **0.00** | **0.00** | **0.00** | **0.00** | **0.04** | **0.04** |
|  | ***Echinophyllia*** | **Total** | **0.00** | **0.00** | **0.00** | **0.00** | **0.01** | **0.00** | **0.00** | **0.00** | **0.00** | **0.00** | **0.00** | **0.00** | **0.00** | **0.00** |
|  |  | *Echinophyllia aspera* | - | - | - | - | 0.01 | - | - | - | - | - | - | - | - | - |
|  |  |  |  |  |  |  |  |  |  |  |  |  |  |  |  |  |
|  | ***Lobophyllia*** | **Total** | **0.00** | **0.00** | **0.00** | **0.00** | **0.00** | **0.00** | **0.00** | **0.00** | **0.00** | **0.00** | **0.00** | **0.00** | **0.01** | **0.01** |
|  |  | other species | - | - | - | - | - | - | - | - | - | - | - | - | 0.01 | 0.01 |
|  |  |  |  |  |  |  |  |  |  |  |  |  |  |  |  |  |
|  | ***Symphyllia*** | **Total** | **0.00** | **0.00** | **0.00** | **0.00** | **0.00** | **0.00** | **0.00** | **0.00** | **0.00** | **0.00** | **0.00** | **0.00** | **0.03** | **0.03** |
|  |  | *Symphyllia radians* | - | - | - | - | - | - | - | - | - | - | - | - | 0.01 | 0.01 |
|  |  | *Symphyllia recta* | - | - | - | - | - | - | - | - | - | - | - | - | 0.01 | 0.01 |
|  |  |  |  |  |  |  |  |  |  |  |  |  |  |  |  |  |
| **Merulinidae** |  | **Total** | **0.55** | **0.53** | **0.57** | **0.59** | **0.66** | **0.63** | **0.67** | **0.51** | **0.57** | **0.50** | **0.55** | **0.50** | **0.55** | **0.55** |
|  | ***Barabattoia*** | **Total** | **0.00** | **0.00** | **0.00** | **0.01** | **0.00** | **0.00** | **0.00** | **0.00** | **0.00** | **0.00** | **0.00** | **0.00** | **0.00** | **0.00** |
|  |  | *Barabattoia amicorum* | - | - | - | 0.01 | - | - | - | - | - | - | - | - | - | - |
|  |  |  |  |  |  |  |  |  |  |  |  |  |  |  |  |  |
|  | ***Cyphastrea*** | **Total** | **0.10** | **0.07** | **0.10** | **0.10** | **0.10** | **0.10** | **0.09** | **0.06** | **0.08** | **0.08** | **0.11** | **0.11** | **0.11** | **0.13** |
|  |  | other species | 0.10 | 0.07 | 0.10 | 0.10 | 0.10 | 0.10 | 0.09 | 0.06 | 0.08 | 0.08 | 0.11 | 0.11 | 0.11 | 0.13 |
|  |  |  |  |  |  |  |  |  |  |  |  |  |  |  |  |  |
|  | ***Dipsastraea*** | **Total** | **0.13** | **0.15** | **0.15** | **0.15** | **0.20** | **0.14** | **0.14** | **0.11** | **0.14** | **0.11** | **0.13** | **0.13** | **0.11** | **0.11** |
|  |  | *Dipsastraea favus* | - | - | - | 0.01 | 0.01 | - | - | - | - | - | - | - | - | - |
|  |  | *Dipsastraea pallida* | 0.01 | 0.03 | 0.03 | 0.01 | 0.06 | 0.03 | 0.02 | 0.01 | 0.01 | 0.01 | 0.01 | 0.01 | 0.01 | 0.01 |
|  |  | *Dipsastraea veroni* | 0.03 | 0.04 | 0.04 | 0.04 | 0.03 | 0.01 | 0.02 | 0.01 | - | - | - | - | - | - |
|  |  | other species | 0.08 | 0.09 | 0.08 | 0.08 | 0.10 | 0.10 | 0.11 | 0.09 | 0.13 | 0.10 | 0.11 | 0.11 | 0.10 | 0.10 |
|  |  |  |  |  |  |  |  |  |  |  |  |  |  |  |  |  |
|  | ***Favites*** | **Total** | **0.14** | **0.12** | **0.11** | **0.13** | **0.14** | **0.17** | **0.16** | **0.13** | **0.11** | **0.11** | **0.11** | **0.11** | **0.11** | **0.13** |
|  |  | *Favites abdita* | 0.03 | 0.03 | 0.03 | 0.03 | 0.03 | 0.06 | 0.03 | 0.04 | 0.03 | 0.01 | 0.01 | 0.01 | 0.01 | 0.01 |
|  |  | *Favites halicora* | 0.03 | 0.01 | 0.01 | 0.01 | 0.01 | 0.03 | 0.03 | 0.03 | 0.01 | - | - | - | 0.01 | 0.01 |
|  |  | other species | 0.08 | 0.08 | 0.07 | 0.08 | 0.10 | 0.08 | 0.11 | 0.06 | 0.07 | 0.10 | 0.10 | 0.10 | 0.08 | 0.10 |
|  |  |  |  |  |  |  |  |  |  |  |  |  |  |  |  |  |
|  | ***Echinopora*** | **Total** | **0.00** | **0.01** | **0.00** | **0.00** | **0.01** | **0.00** | **0.00** | **0.00** | **0.00** | **0.00** | **0.00** | **0.00** | **0.00** | **0.00** |
|  |  | *Echinopora lamellosa* | **-** | 0.01 | **-** | **-** | 0.01 | **-** | **-** | **-** | **-** | **-** | **-** | **-** | **-** | **-** |
|  |  |  |  |  |  |  |  |  |  |  |  |  |  |  |  |  |
|  | ***Goniastrea*** | **Total** | **0.06** | **0.05** | **0.07** | **0.06** | **0.07** | **0.07** | **0.09** | **0.07** | **0.07** | **0.07** | **0.07** | **0.06** | **0.07** | **0.07** |
|  |  | *Goniastrea aspera* | 0.01 | 0.01 | 0.01 | 0.01 | 0.01 | 0.03 | 0.03 | 0.03 | 0.03 | 0.03 | 0.03 | 0.03 | 0.03 | 0.03 |
|  |  | *Goniastrea pectinata* | 0.03 | 0.03 | 0.04 | 0.04 | 0.06 | 0.04 | 0.06 | 0.04 | 0.04 | 0.04 | 0.04 | 0.03 | 0.04 | 0.04 |
|  |  | *Goniastrea retiformis* | 0.01 | 0.01 | 0.01 | - | - | - | - | - | - | - | - | - | - | - |
|  |  |  |  |  |  |  |  |  |  |  |  |  |  |  |  |  |
|  | ***Oulophyllia*** | **Total** | **0.04** | **0.08** | **0.06** | **0.06** | **0.04** | **0.07** | **0.07** | **0.04** | **0.06** | **0.04** | **0.06** | **0.06** | **0.07** | **0.06** |
|  |  | *Oulophyllia crispa* | 0.04 | 0.08 | 0.06 | 0.06 | 0.04 | 0.07 | 0.07 | 0.04 | 0.06 | 0.04 | 0.06 | 0.06 | 0.07 | 0.06 |
|  |  |  |  |  |  |  |  |  |  |  |  |  |  |  |  |  |
|  | ***Phymastrea*** | **Total** | **0.06** | **0.03** | **0.06** | **0.08** | **0.08** | **0.07** | **0.09** | **0.06** | **0.08** | **0.07** | **0.06** | **0.04** | **0.06** | **0.04** |
|  |  | *Phymastrea annuligera* | - | - | - | - | - | - | - | - | - | - | - | - | 0.01 | - |
|  |  | *Phymastrea valenciennesi* | 0.01 | - | 0.01 | 0.03 | 0.03 | 0.03 | 0.03 | 0.03 | 0.03 | 0.03 | 0.03 | 0.01 | 0.01 | 0.01 |
|  |  | other species | 0.04 | 0.03 | 0.04 | 0.06 | 0.06 | 0.04 | 0.06 | 0.03 | 0.06 | 0.04 | 0.03 | 0.03 | 0.03 | 0.03 |
|  |  |  |  |  |  |  |  |  |  |  |  |  |  |  |  |  |
|  | ***Platygyra*** | **Total** | **0.03** | **0.03** | **0.03** | **0.00** | **0.01** | **0.01** | **0.03** | **0.04** | **0.03** | **0.01** | **0.01** | **0.00** | **0.01** | **0.01** |
|  |  | *Platygyra lamellina* | - | - | - | - | 0.01 | 0.01 | 0.03 | 0.03 | 0.03 | 0.01 | 0.01 | - | - | - |
|  |  | *Platygyra pini* | 0.01 | 0.01 | 0.01 | - | - | - | - | 0.01 | - | - | - | - | - | - |
|  |  | other species | 0.01 | 0.01 | 0.01 | - | - | - | - | - | - | - | - | - | 0.01 | 0.01 |
|  |  |  |  |  |  |  |  |  |  |  |  |  |  |  |  |  |
| **Milleporidae** |  | **Total** | **0.07** | **0.10** | **0.10** | **0.04** | **0.04** | **0.10** | **0.00** | **0.00** | **0.00** | **0.00** | **0.00** | **0.03** | **0.06** | **0.06** |
|  | ***Millepora*** | **Total** | **0.07** | **0.10** | **0.10** | **0.04** | **0.04** | **0.10** | **0.00** | **0.00** | **0.00** | **0.00** | **0.00** | **0.03** | **0.06** | **0.06** |
|  |  | *Millepora exaesa* | 0.03 | 0.01 | 0.03 | - | - | - | - | - | - | - | - | 0.01 | 0.01 | 0.01 |
|  |  | *Millepora platyphylla* | 0.01 | 0.01 | 0.01 | 0.01 | 0.01 | 0.01 | - | - | - | - | - | - | - | - |
|  |  | *Millepora tenella* | - | 0.01 | - | 0.01 | 0.01 | 0.03 | - | - | - | - | - | - | - | - |
|  |  | other species | 0.03 | 0.07 | 0.06 | 0.01 | 0.01 | 0.06 | - | - | - | - | - | 0.01 | 0.04 | 0.04 |
|  |  |  |  |  |  |  |  |  |  |  |  |  |  |  |  |  |
| **Pocilloporidae** | | **Total** | **0.22** | **0.23** | **0.24** | **0.04** | **0.03** | **0.04** | **0.00** | **0.01** | **0.01** | **0.01** | **0.06** | **0.06** | **0.08** | **0.06** |
|  | ***Palauastrea*** | **Total** | **0.00** | **0.00** | **0.01** | **0.00** | **0.00** | **0.00** | **0.00** | **0.00** | **0.00** | **0.00** | **0.00** | **0.00** | **0.00** | **0.00** |
|  |  | *Palauastrea ramosa* | **-** | **-** | 0.01 | **-** | **-** | **-** | **-** | **-** | **-** | **-** | **-** | **-** | **-** | **-** |
|  |  |  |  |  |  |  |  |  |  |  |  |  |  |  |  |  |
|  | ***Pocillopora*** | **Total** | **0.10** | **0.09** | **0.06** | **0.01** | **0.01** | **0.01** | **0.00** | **0.00** | **0.00** | **0.00** | **0.03** | **0.03** | **0.06** | **0.04** |
|  |  | *Pocillopora damicornis* | 0.07 | 0.08 | 0.04 | 0.01 | 0.01 | 0.01 | - | - | - | - | 0.03 | 0.03 | 0.06 | 0.04 |
|  |  | *Pocillopora eydouxi* | 0.01 | 0.01 | 0.01 | - | - | - | - | - | - | - | - | - | - | - |
|  |  | other species | 0.01 | - | - | - | - | - | - | - | - | - | - | - | - | - |
|  |  |  |  |  |  |  |  |  |  |  |  |  |  |  |  |  |
|  | ***Seriatopora*** | **Total** | **0.00** | **0.03** | **0.01** | **0.01** | **0.00** | **0.01** | **0.00** | **0.01** | **0.00** | **0.00** | **0.00** | **0.00** | **0.00** | **0.00** |
|  |  | *Seriatopora hystrix* | - | 0.03 | 0.01 | 0.01 | - | 0.01 | - | 0.01 | - | - | - | - | - | - |
|  |  |  |  |  |  |  |  |  |  |  |  |  |  |  |  |  |
|  | ***Stylophora*** | **Total** | **0.13** | **0.11** | **0.15** | **0.01** | **0.01** | **0.01** | **0.00** | **0.00** | **0.01** | **0.01** | **0.03** | **0.03** | **0.03** | **0.01** |
|  |  | *Stylophora pistillata* | 0.13 | 0.11 | 0.15 | 0.01 | 0.01 | 0.01 | - | - | 0.01 | 0.01 | 0.03 | 0.03 | 0.03 | 0.01 |
|  |  |  |  |  |  |  |  |  |  |  |  |  |  |  |  |  |
| **Poritidae** |  | **Total** | **0.27** | **0.30** | **0.28** | **0.29** | **0.29** | **0.28** | **0.27** | **0.20** | **0.27** | **0.27** | **0.31** | **0.27** | **0.27** | **0.27** |
|  | ***Porites*** | **Total** | **0.27** | **0.30** | **0.28** | **0.29** | **0.29** | **0.28** | **0.27** | **0.20** | **0.27** | **0.27** | **0.31** | **0.27** | **0.27** | **0.27** |
|  |  | *Porites cylindrica* | 0.06 | 0.06 | 0.06 | 0.04 | 0.03 | 0.04 | 0.03 | 0.01 | 0.01 | 0.01 | 0.03 | 0.01 | 0.01 | 0.01 |
|  |  | *Porites lutea* | 0.03 | 0.05 | 0.04 | 0.04 | 0.03 | 0.03 | 0.03 | 0.03 | 0.03 | 0.03 | 0.03 | 0.03 | 0.03 | 0.03 |
|  |  | *Porites rus* | - | - | - | 0.01 | 0.01 | 0.01 | - | - | - | - | - | - | - | - |
|  |  | other species | 0.18 | 0.18 | 0.18 | 0.20 | 0.22 | 0.20 | 0.21 | 0.16 | 0.22 | 0.22 | 0.25 | 0.22 | 0.22 | 0.22 |
|  |  |  |  |  |  |  |  |  |  |  |  |  |  |  |  |  |
| **Siderastreidae** | | **Total** | **0.01** | **0.05** | **0.04** | **0.06** | **0.06** | **0.07** | **0.03** | **0.03** | **0.04** | **0.06** | **0.06** | **0.06** | **0.07** | **0.07** |
|  | ***Psammocora*** | **Total** | **0.01** | **0.05** | **0.04** | **0.06** | **0.06** | **0.07** | **0.03** | **0.03** | **0.04** | **0.06** | **0.06** | **0.06** | **0.07** | **0.07** |
|  |  | *Psammocora contigua* | - | 0.01 | - | - | - | - | - | - | - | - | - | - | - | - |
|  |  | *Psammocora profundacella* | 0.01 | 0.04 | 0.04 | 0.06 | 0.06 | 0.07 | 0.03 | 0.03 | 0.04 | 0.06 | 0.06 | 0.06 | 0.07 | 0.07 |
|  |  |  |  |  |  |  |  |  |  |  |  |  |  |  |  |  |
| ***Incertae sedis**** |  | **Total** | **0.10** | **0.17** | **0.15** | **0.15** | **0.20** | **0.15** | **0.15** | **0.15** | **0.17** | **0.13** | **0.13** | **0.15** | **0.17** | **0.17** |
|  | ***Leptastrea*** |  | **0.07** | **0.12** | **0.11** | **0.11** | **0.14** | **0.11** | **0.10** | **0.12** | **0.13** | **0.08** | **0.10** | **0.11** | **0.13** | **0.14** |
|  |  | *Leptastrea purpurea* | 0.04 | 0.05 | 0.07 | 0.06 | 0.08 | 0.06 | 0.04 | 0.06 | 0.07 | 0.03 | 0.03 | 0.04 | 0.06 | 0.06 |
|  |  | other species | 0.03 | 0.07 | 0.04 | 0.06 | 0.06 | 0.06 | 0.06 | 0.06 | 0.06 | 0.06 | 0.07 | 0.07 | 0.07 | 0.08 |
|  |  |  |  |  |  |  |  |  |  |  |  |  |  |  |  |  |
|  | ***Oulastrea*** | **Total** | **0.03** | **0.05** | **0.04** | **0.04** | **0.06** | **0.04** | **0.04** | **0.03** | **0.04** | **0.04** | **0.03** | **0.04** | **0.04** | **0.03** |
|  |  | *Oulastrea crispata* | 0.03 | 0.05 | 0.04 | 0.04 | 0.06 | 0.04 | 0.04 | 0.03 | 0.04 | 0.04 | 0.03 | 0.04 | 0.04 | 0.03 |

The values indicate abundance of species (total number/m^2^). The absence of speceis is shown by "-". The taxonomic research of family *Incertae sedis* (*) is now in progress [1].

[1] Budd AN, Fukami H, Smith ND, Knowlton N (2012) Taxonomic classification of the reef coral family Mussidae (Cnidaria: Anthozoa: Scleractinia). Zoological Journal of the Linnean Society 166: 465–529.
