# Supplementary material for: Species-Specific Responses of Corals to Bleaching Events on Anthropogenically Turbid Reefs on Okinawa Island, Japan, over a 15-year Period (1995–2009)
Source: PLoS One. 2013 Apr 2;8(4):e60952. doi: 10.1371/journal.pone.0060952 (PMC3614915; doi:10.1371/journal.pone.0060952)
Supplement: Table S4 — Result of Kruskal–Wallis test of temporal change in the coral genera from 1995 to 2009 at Okinawa Island. (DOC) [file pone.0060952.s004.doc]

| ***Coral genera*** | ***Χ^2^*** | ***P* value** |
| --- | --- | --- |
| *Acropora* | 39.1 | ** |
| *Anacropora* |  | NS |
| *Astreopora* |  | NS |
| *Barabattoia* |  | NS |
| *Cyphastrea* |  | NS |
| *Dipsastraea* |  | NS |
| *Echinophyllia* |  | NS |
| *Echinopora* |  | NS |
| *Favites* |  | NS |
| *Galaxea* |  | NS |
| *Goniastrea* |  | NS |
| *Leptastrea* |  | NS |
| *Millepora* | 29.8 | * |
| *Montipora* |  | NS |
| *Oulastrea* |  | NS |
| *Oulophyllia* |  | NS |
| *Palauastrea* |  | NS |
| *Pavona* |  | NS |
| *Phymastrea* |  | NS |
| *Platygyra* |  | NS |
| *Pocillopora* | 36.2 | ** |
| *Porites* |  | NS |
| *Psammocora* |  | NS |
| *Seriatopora* |  | NS |
| *Stylocoeniella* | 43.7 | ** |
| *Stylophora* | 70.6 | ** |
| *Turbinaria* |  | NS |
| NS: not significant; *P < 0.01; **P < 0.001 | | |
