# Supplementary material for: Species-Specific Responses of Corals to Bleaching Events on Anthropogenically Turbid Reefs on Okinawa Island, Japan, over a 15-year Period (1995–2009)
Source: PLoS One. 2013 Apr 2;8(4):e60952. doi: 10.1371/journal.pone.0060952 (PMC3614915; doi:10.1371/journal.pone.0060952)
Supplement: Table S5 — Result of Kruskal–Wallis test of temporal change in the coral species from 1995 to 2009 at Okinawa Island. Dominant species of Acropora, Dipsastraea, Goniastrea, Leptastrea, Millepora, Montipora, Oulastrea, Pocillopora, Porites, Stylophora, and Stylocoeniella were selected for the analysis. (DOC) [file pone.0060952.s005.doc]

| ***Coral species*** | ***Growth form*†** | ***Χ^2^*** | ***P* value** |
| --- | --- | --- | --- |
| *Acropora acuminata* | branching |  | NS |
| *Acropora aspera* | branching | 26.2 | * |
| *Acropora austera* | branching |  | NS |
| *Acropora brueggemanni* | branching |  | NS |
| *Acropora clathrata* | tabular |  | NS |
| *Acropora digitifera* | tabular | 34.2 | *** |
| *Acropora divaricata* | tabular |  | NS |
| *Acropora florida* | tabular |  | NS |
| *Acropora gemmifera* | tabular |  | NS |
| *Acropora humilis* | tabular |  | NS |
| *Acropora hyacinthus/ cytherea* | tabular | 34.4 | *** |
| *Acropora intermedia/ muricata* | branching | 26.8 | * |
| *Acropora loripes* | tabular |  | NS |
| *Acropora microphthalma* | branching |  | NS |
| *Acropora monticulosa* | tabular |  | NS |
| *Acropora nana* | tabular |  | NS |
| *Acropora nasuta* | tabular |  | NS |
| *Acropora secale* | tabular |  | NS |
| *Acropora selago* | tabular |  | NS |
| *Acropora subulata* | tabular |  | NS |
| *Acropora tenuis* | tabular | 31.0 | ** |
| *Acropora valida* | tabular |  | NS |
| *Acropora yongei* | branching |  | NS |
| *Dipsastraea pallida/ speciosa/ favus* | massive |  | NS |
| *Goniastrea aspera* | massive |  | NS |
| *Leptastrea purpurea* | massive |  | NS |
| *Millepora exaesa* | massive |  | NS |
| *Millepora platyphylla* | complex |  | NS |
| *Millepora tenella* | branching |  | NS |
| *Millepora* spp. | complex | 32.4 | ** |
| *Montipora digitata* | branching |  | NS |
| *Oulastrea crispata* | massive |  | NS |
| *Pocillopora damicornis* | branching | 26.2 | * |
| *Pocillopora eydouxi* | complex |  | NS |
| *Porites cylindrica* | branching |  | NS |
| *Porites lutea/australiensis* | massive |  | NS |
| *Stylocoeniella guentheri* | complex |  | NS |
| *Stylocoeniella* spp. | complex | 24.8 | * |
| *Stylophora pistillata* | branching | 70.6 | *** |
| †: tabular including corymbose, complex showing a complex form of sub massive and/or encrusting and/or branching | | | |
| NS: not significant; *P < 0.05; **P < 0.01; ***P < 0.001 | | | |
